# Microbiome profile and calprotectin levels as markers of risk of recurrent *Clostridioides difficile* infection

**DOI:** 10.3389/fcimb.2023.1237500

**Published:** 2023-09-13

**Authors:** Silvia Vázquez-Cuesta, Nuria Lozano García, Ana I. Fernández, María Olmedo, Martha Kestler, Luis Alcalá, Mercedes Marín, Javier Bermejo, Francisco Fernández-Avilés Díaz, Patricia Muñoz, Emilio Bouza, Elena Reigadas

**Affiliations:** ^1^ Department of Clinical Microbiology and Infectious Diseases, Hospital General Universitario Gregorio Marañón, Madrid, Spain; ^2^ Instituto de Investigación Sanitaria Gregorio Marañón, Madrid, Spain; ^3^ Biochemistry and Molecular Biology Department, Faculty of Biology, Universidad Complutense de Madrid (UCM), Madrid, Spain; ^4^ Department of Cardiology, Hospital General Universitario Gregorio Marañón, Madrid, Spain; ^5^ Medicine Department, School of Medicine, Universidad Complutense de Madrid (UCM), Madrid, Spain; ^6^ Centro de Investigación Biomédica en red de Enfermedades Respiratorias (CIBERES CB06/06/0058), Madrid, Spain; ^7^ Centro de Investigación Biomédica en red de Enfermedades Cardiovasculares (CIBERCV), Madrid, Spain

**Keywords:** calprotectin, biomarkers, *C. difficile*, microbiome, R-CDI, 16S rRNA, prediction model

## Abstract

**Introduction:**

Clostridioides difficile infection (CDI) is the main cause of nosocomial diarrhoea in developed countries. Recurrent CDI (R-CDI), which affects 20%-30% of patients and significantly increases hospital stay and associated costs, is a key challenge. The main objective of this study was to explore the role of the microbiome and calprotectin levels as predictive biomarkers of R-CDI.

**Methods:**

We prospectively (2019-2021) included patients with a primary episode of CDI. Clinical data and faecal samples were collected. The microbiome was analysed by sequencing the hypervariable V4 region of the 16S rRNA gene on an Illumina Miseq platform.

**Results:**

We enrolled 200 patients with primary CDI, of whom 54 developed R-CDI and 146 did not. We analysed 200 primary samples and found that Fusobacterium increased in abundance, while Collinsella, Senegalimassilia, Prevotella and Ruminococcus decreased in patients with recurrent versus non-recurrent disease. Elevated calprotectin levels correlated significantly with R-CDI (p=0.01). We built a risk index for R-CDI, including as prognostic factors age, sex, immunosuppression, toxin B amplification cycle, creatinine levels and faecal calprotectin levels (overall accuracy of 79%).

**Discussion:**

Calprotectin levels and abundance of microbial genera such as Fusobacterium and Prevotella in primary episodes could be useful as early markers of R-CDI. We propose a readily available model for prediction of R-CDI that can be applied at the initial CDI episode. The use of this tool could help to better tailor treatments according to the risk of R-CDI.

## 1 Introduction

The clinical severity of *Clostridioides difficile* infection ranges from asymptomatic, mild, moderate and severe episodes and pseudomembranous colitis to toxic megacolon, sepsis or death (Rupnik et al., 2009).

One of the current challenges associated with CDI is recurrent CDI (R-CDI), which usually affects 20%-30% of patients with a first CDI infection and increases to 40%-65% after a first R-CDI episode (Barbut et al., 2000; Leffler and Lamont, 2015; Larrainzar-Coghen et al., 2016; Smits et al., 2016).

The costs associated with R-CDI in the United States reach $94,209 per year for patients with 1 R-CDI episode, increasing to $207,733 per year for patients with 3 or more R-CDI, with a length of stay ranging from 8.3 to 17.9 days (Feuerstadt et al., 2020). In Europe each episode of R-CDI involves an average cost of €40,941 and a length of stay of 55 days; these costs are 4-fold higher and with a 2.5-fold longer stay than for a primary CDI episode (Wingen-Heimann et al., 2022).

The main risk factors associated with R-CDI include advanced age, hospital exposure, treatment with proton pump inhibitors, prior antibiotic treatment and initial disease severity (Eyre et al., 2012; Lupse et al., 2013; Alrahmany et al., 2021).

The imbalance of gut’s microbial community, known as gut microbiota dysbiosis, is strongly linked to the risk of CDI, especially that resulting from the action of antibiotics (Buffie et al., 2012; Theriot et al., 2016). However, very few papers in the literature investigate the association between gut microbiota factors and the risk of R-CDI, and those that do include a low number of patients and report very disparate results (Khanna et al., 2016; Seekatz et al., 2016; Pakpour et al., 2017; Dawkins et al., 2022).Khanna et al. included 88 patients with CDI, 22 of them developed R-CDI; Seekatz et al. included 93 patients with CDI and 32 developed R-CDI, and Dawkins et al. included 53 patients with CDI and 19 developed R-CDI, all they found no significant differences in either alpha diversity or beta diversity. Pakpour et al. included 31 patients with CDI, of them 11 developed R-CDI, they found significant differences in alpha diversity and beta diversity. Because of the close relationship between gut microbiota and *C. difficile*, treatment with faecal microbiota transplantation for R-CDI to restore patients’ gut microbiota and prevent future recurrences is already included in IDSA/SHEA guidelines (McDonald et al., 2018). For this reason, clinical trials are now investigating the use of faecal microbiota transplantation in first episodes to reduce the risk of recurrence (Kates et al., 2020).

New CDI treatments for reducing recurrences include fidaxomicin, bezlotoxumab, and SER-109 (Escudero-Sánchez et al., 2020; Dubberke et al., 2022; Feuerstadt et al., 2022; Johnson et al., 2022; Tashiro et al., 2022).Both fidaxomicin and bezlotoxumab involve reducing damage to the gut microbiota in CDI patients, which has been shown to result in lower recurrence rates. SER-109 is an orally administered faecal microbiota treatment, helping microbiota recovery to prevent possible recurrences, just like the faecal microbiota transplants that have been performed for years. However, new CDI treatments are costly, and recent European guidelines suggest applying a risk stratification strategy in cases of economic restraints (van Prehn et al., 2021). Many attempts have been made to try to identify or profile patients at high risk for CDI (van Rossen et al., 2022), to date, no reliable objective markers to help predict who is at increased risk of R-CDI have been identified.

There are different markers of inflammation that can be measured directly in faeces, including faecal leukocyte analysis, in which blood is drawn from the patient, the leukocytes are labelled with a radioisotope and reintroduced to the patient. Stool samples are then collected and quantified for labelled leukocytes (Stathaki et al., 2009). Its main drawback is that it is invasive and exposes patients to radiation. Other methods involve measuring leukocyte proteins such as myeloperoxidase, lysozyme, elastase, S100A12, lactoferrin and calprotectin in the stool (Gonzalez et al., 2015; Lehmann et al., 2015; Galgut et al., 2018). Of these, calprotectin has been shown to be the most stable in faeces (Gonzalez et al., 2015) and is therefore the most commonly used in clinical practice, hence we included faecal calprotectin in our analyses.

Calprotectin is a protein secreted mainly by neutrophils, but also by macrophages, monocytes and dendritic cells (Odink et al., 1987; Edgeworth et al., 1991; Kumar et al., 2003). It is known to be a good marker of intestinal inflammation, can be obtained non-invasively and is stable at room temperature (Xu and Geczy, 2000; Lasson et al., 2015).Several studies have examined the role of calprotectin as a marker of severity of CDI with calprotectin levels being higher the more severe the CDI episode (Peretz et al., 2016; Kim et al., 2017; Suarez-Carantoña et al., 2021) and as a marker of CDI itself with higher levels of calprotectin in patients with CDI than in patients without CDI (Popiel et al., 2015; Barbut et al., 2017).

The high associated health and economic burden of R-CDI calls for the development of novel strategies by which R-CDI can be prevented in susceptible patients (Heimann et al., 2018; Reigadas Ramirez and Bouza, 2018). Profiling differences between gut microbiota and calprotectin levels in patients with CDI could help to predict which patients are more likely to experience R-CDI.

The main objective of this study was to explore the role of the microbiome and calprotectin as predictive biomarkers of R-CDI.

## 2 Methods

### 2.1 Setting, design, and study population

This study was carried out at Hospital General Universitario Gregorio Marañón in Madrid (Spain), a tertiary university hospital with 1,350 beds. Toxigenic *C. difficile* is routinely investigated in all diarrhoeic stool samples from patients older than 2 years. The microbiology laboratory receives samples both from the hospital itself and from 13 outpatient centres in the same area.

We conducted a prospective study from January 2019 to April 2021. We enrolled patients over 2 years old with a positive test result for toxigenic *C. difficile* and met the clinical criteria for CDI. We excluded patients younger than 2 years old because they have a high colonisation rate, which in many cases makes it difficult to determine the true cause of diarrhoea, in infants less than 7 days are 15%, in infants between 6 and 12 months 41% and 22% in 2 year old (Tougas et al., 2021). It appears that the infants’ gut is immune to the effects of toxins A and B although the mechanism is unknown, very few cases of CDI have been described in infants under 2 years of age (Jangi and Lamont, 2010). We collected clinical data and faecal samples from all patients. Patients were classified into 2 groups: patients with a primary CDI episode who did not subsequently develop recurrent CDI (non-recurrent) and patients with a primary CDI episode who subsequently developed recurrent CDI (recurrent).

### 2.2 Definitions

An episode of CDI was defined as the presence of a positive toxigenic CDI test result, together with diarrhoea (≥3 unformed stools in 24 h) or findings of pseudomembranous colitis by colonoscopy, following the definitions set out in the guidelines of the Society for Healthcare Epidemiology of America (SHEA) and the Infectious Diseases Society of America (IDSA) (McDonald et al., 2018).

CDI is considered recurrent when it recurs within 8 weeks after a previous episode, provided the symptoms from the previous episode resolved after completion of the initial treatment (van Prehn et al., 2021). Having new symptoms and a positive sample after 60 days was considered a new episode.

Defined daily dose (DDD) is defined in the “WHO Collaborating Centre for Drug Statistics Methodology” as the assumed average maintenance dose per day in the case of a drug used for its main indication in adults (WHO, 2021).

We classified antimicrobial drugs as anaerobic, as previously described by Mulder et al, “We also classified the antimicrobial drugs in antimicrobial drugs with anaerobic activity (consisting of combinations of penicillins, including beta-lactamase inhibitors (J01CR), lincosamides (J01 FF) and imidazole derivatives (metronidazole) (J01XD): anaerobic+)…” (Mulder et al., 2020).

The severity of the CDI episode was defined according to the SHEA and IDSA guidelines (McDonald et al., 2018).

Death was considered CDI-related when there were no other attributable causes and/or it occurred within 10 days after the diagnosis of CDI and/or was due to known complications of CDI.

As for microbiome-related definitions, we considered richness as the number of different species found in a sample (Whittaker et al., 2001). Evenness (Pielou index) was defined as the degree to which different species are similar or uniform in abundance. Diversity indicated the degree of species richness and abundance, where alpha diversity is referred to the diversity within an individual -Inverse Simpson index: an indication of the richness in a community with the same evenness; and Shannon index: takes into account the number of species living in a habitat (richness) and their relative abundance (evenness)- and beta diversity referred to the difference in diversity between individuals (Whittaker et al., 2001).

We considered as microbiota-related diseases the following: cholelithiasis, colorectal cancer, hepatic encephalopathy, idiopathic constipation, inflammatory bowel disease, irritable bowel syndrome, familial Mediterranean fever, gastric lymphoma or carcinoma, arthritis, asthma, atopy, dermatitis, psoriasis, autoimmune disease, fatigue syndrome, diabetes mellitus, hypercholesterolaemia, idiopathic thrombocytopenic purpura, myocardial ischaemia, metabolic syndrome, behavioural disorders, multiple sclerosis, myoclonus dystonia, non-alcoholic fatty liver disease, oxalate kidney stones and Parkinson’s disease.

### 2.3 Detection of toxigenic *C. difficile*

Samples were processed using a rapid detection kit for toxigenic *C. difficile*. This rapid test consists of glutamate dehydrogenase antigen and toxins A and B detection by immunochromatography (C Diff Quik-Chek Complete assay, TechLab, Blacksburg, VA, USA) (Sensitivity 90.5%, specificity 93.1%, predictive positive value 76.4%, predictive negative value 97.6% in comparison with bacterial culture) and a real-time polymerase chain reaction (PCR) assay of the *C. difficile* toxin B gene (Xpert.*C. difficile* Assay, GeneXpert, Cepheid, Sunnyvale, CA, USA) (Sensitivity 98.79%, specificity 90.82%, predictive positive value 56.58%, predictive negative value 99.83% in comparison with bacterial culture).

In addition, all samples were cultured on *C. difficile* selective agar (bioMeriéux, Marcy l’Etoile, France). Suspected toxigenic *C. difficile* colonies were confirmed using immunochromatography (C Diff Quik-Chek Complete assay, TechLab, Blacksburg, VA, USA).

### 2.4 Calprotectin assay

Calprotectin levels were measured using EDITM Quantitative Faecal Calprotectin ELISA (Measurement range: 1 – 2000 µg/g stool) (Epitope Diagnostics, Inc, San Diego, CA, USA), according to the manufacturers’ instructions.

### 2.5 Clinical data

The demographic data collected included age and sex. Regarding clinical data, the underlying conditions were recorded using the McCabe and Jackson score for prognosis of underlying diseases. The McCabe score was used to categorise the risk of death by underlying comorbidities. The 3 categories were rapidly fatal (within 1 year), ultimately fatal (within 5 years) and non-fatal (>5 years) (Jackson and McCabe, 1962); comorbidity was graded according to the Charlson comorbidity index (Charlson et al., 1987). The Charlson comorbidity index is one of the most extensively used comorbidity indices (Huang et al., 2014). This index consists of 19 conditions, each of which is assigned a weighting of 1, 2, 3 or 6 based on the adjusted hazard ratio for each comorbidity. The sum of the weighted scores gives the total score (Yurkovich et al., 2015).

Other clinical data collected included antibiotic treatment, proton pump inhibitor use, nasogastric tube use, mechanical ventilation, surgery and chemotherapy or radiotherapy in the month prior to diagnosis of CDI. Regarding antibiotic consumption, the DDDs for each antibiotic were recorded. For the CDI episode, data on severity of the episode, treatment received, treatment failure, recurrence, mortality and CDI-related mortality were recorded.

### 2.6 Sample processing

Immediately upon receipt, the faecal samples were homogenized, aliquoted and stored at −80°C until the day of analysis. Total DNA was extracted from faecal samples using the Qiagen Fast QiaAmp DNA stool mini kit (QIAGEN, Valencia, CA, USA) according to the manufacturer’s protocol with the inclusion of a physical lysis step. The sample was lysed twice at 6.5m/s for 45 seconds in FastPrep-24 (MPBio, Derby, UK) with lysis matrix tubes E (MPBio, Derby, UK). The hypervariable V4 region of the 16S rRNA gene was amplified by PCR, with 515-806 primers (515:GTGCCAGCMGCCGCGGTAA, 806:GGACTACHVGGGTWTCTAAT) tailed with sequences to incorporate Illumina flow cell adapters and indexing barcodes (Illumina, San Diego, CA, USA). PCR amplification program was 1 cycle of 98°C 30 seconds; 25 cycles of 98°C 10 seconds, 60°C 20 seconds, 72°C 20 seconds; 1 cycle of 72°C 2 minutes. Q5® High-Fidelity 2X Master Mix (New England Biolabs, Ipswich, MA, USA)

Primer dimers and low-molecular-weight products were removed using Agencourt Ampure Beads (Beckman Coulter, Spain). Samples were quantified and quality was checked for amplicon size using the 4200 TapeStation (Agilent Technologies, Santa Clara, CA, USA). Amplicons were equimolar pooled and sequenced (2 × 250) on an Illumina Miseq platform (Illumina, San Diego, CA, USA) according to standard protocols.

### 2.7 Data analysis

The raw data were pre-processed, grouped by operational taxonomic units (OTUs) with 97% similarity and taxonomically classified using MOTHUR software (Patrick D. Schloss, PhD, © 2019, Michigan, USA) and SILVA and RDP databases. Analyses of species richness (OTUs observed), evenness (Pielou index), alpha diversity (Shannon index) and beta diversity (Bray-Curtys distance, unweighted unifrac distance) were performed with MOTHUR and R software (R Core Team, 2021, Vienna, Austria).

The statistical analyses were performed using R (R Core Team, 2021, Vienna, Austria). Frequencies were calculated for qualitative variables, and proportions were calculated with their 95% confidence interval (CI) following a binomial distribution. For quantitative variables, the median and interquartile range (IQR) or mean and standard deviation (SD) were calculated. Microbiota analyses were performed with R using the packages phyloseq, microbiome, microbiomeStat, vegan, DESeq2 and microeco.

The MaAslin2 (Multivariate microbial Association by Linear models) library was used to study the relationship between microbiota and clinical variables (Mallick et al., 2021). Differences between groups were determined using the χ^2^ test. Continuous variables were compared using the *t* test or the Mann-Whitney test (when a normal distribution could not be assumed). The normality of the distribution of continuous variables was assessed using the Kolmogorov-Smirnov test with the Lilliefors correction. A multivariate logistic regression model was used to assess predictors of R-CDI. The odds ratio (OR) and 95% CI were calculated. A p value <0.05 was considered significant. All significant variables in the bivariate study were included in the multivariate model, in the multivariate study a logistic regression was performed in which the weight of each variable was scored according to its odds ratio.

### 2.8 Ethical issues

This study was approved by the Ethics Committee of Hospital General Universitario Gregorio Marañón in Madrid (number MICRO.HGUGM.2016-029).

Participants’ informed consent was obtained before enrolment. All patients included in the study were given the sufficient time to make the decision to participate and understood each of the components of the study. Patients were also informed about their rights of access, rectification, cancellation and opposition.

### 2.9 Data availability

The data for this study have been deposited in the European Nucleotide Archive (ENA) at EMBL-EBI under accession number PRJEB57947 (https://www.ebi.ac.uk/ena/browser/view/PRJEB57947).

## 3 Results

During the study period, there were 1308 samples positive for toxigenic *C. difficile* from 906 patients. We obtained informed consent and a valid sample from 227 patients who had their first episode of CDI. Of these, 24 patients died before the end of the 2-month recurrence period follow-up, and 3 patients were lost to follow-up. We selected for analysis only samples from the patient’s first episode. Finally, we had 200 primary samples belonging to 200 patients, of whom 54 developed R-CDI and 146 did not.

### 3.1 Demographic and clinical characteristics

The median age of the patients was 67 years for those with non-recurrent disease and 78.50 years for those with recurrent disease. In both groups, there were more females than males (non-recurrent 54.8% [80/146]; recurrent 74.1% [40/54]; p = 0.013).

The most common underlying diseases were cardiovascular, metabolic, endocrine, nephrourological and gastrointestinal diseases (**Table 1**). The median Charlson comorbidity index was 4 (IQR: 2-6) in the non-recurrent group and 3.50 (IQR: 3-6) in the recurrent group (p= 0.256). Both groups contained a high percentage of patients with underlying diseases related to alterations of the microbiota, although this difference was not statistically significant (non-recurrent 65.8% [96/146]; recurrent 75.9% [41/54]; (p=0.169). Within this group of diseases, diabetes mellitus and ischemic heart disease were the most common (**Table 1**). Approximately 36% of all patients were immunocompromised (**Table 1**). Surprisingly, a higher percentage of patients had inflammatory bowel disease in the non-recurrent group (13.5%) than in the recurrent group (2.4%), and a higher percentage of patients had arthritis in the recurrent group (12.2%) than in the non-recurrent group (3.1%) (all p <0.05).

**Table 1:** Clinical characteristics of patients with a primary *Clostridioides difficile* infection episode.

	Non-Rec (n=146)	Rec (n=54)	p value
**AGE** Median (Q1, Q3)	67.00 (55.00, 79.00)	78.50 (70.25, 85.75)	**< 0.001**
**SEX**			**0.013**
Female	80 (54.8%)	40 (74.1%)	
Male	66 (45.2%)	14 (25.9%)	
**ADMITTED PATIENT**	106 (72.6%)	29 (53.7%)	**0.011**
**INSTITUTIONALISED PATIENT**	5 (3.4%)	7 (13.0%)	**0.012**
**DAYS OF HOSPITAL STAY** Median (Q1, Q3)	16.00 (9.00, 36.25)	13.00 (8.00, 27.00)	0.222
**HIV**	6 (4.1%)	2 (3.7%)	0.897
**SOLID ORGAN TRANSPLANT**	20 (13.7%)	7 (13.0%)	0.892
**MALIGNANCY**	30 (20.5%)	13 (24.1%)	0.59
**CARDIOLOGICAL DISEASE**	97 (66.4%)	50 (92.6%)	**< 0.001**
**PULMONARY DISEASE**	32 (21.9%)	15 (27.8%)	0.386
**GASTROINTESTINAL DISEASE**	56 (38.4%)	19 (35.2%)	0.681
**LIVER DISEASE**	40 (27.4%)	8 (14.8%)	0.064
**HAEMATOLOGIC MALIGNANCY**	31 (21.2%)	7 (13.0%)	0.186
**ENDOCRINE DISEASE**	60 (41.1%)	28 (51.9%)	0.174
**METABOLIC DISEASE**	64 (43.8%)	34 (63.0%)	**0.016**
**INFECTIOUS DISEASE**	18 (12.3%)	6 (11.1%)	0.814
**ALLERGIC DISEASE**	3 (2.1%)	0 (0.0%)	0.289
**RHEUMATIC DISEASE**	29 (19.9%)	20 (37.0%)	**0.012**
**NEUROLOGICAL DISEASE**	40 (27.4%)	18 (33.3%)	0.411
**NEPHROUROLOGICAL DISEASE**	53 (36.3%)	27 (50.0%)	0.079
**IMMUNE-MEDIATED DISEASE**	6 (4.1%)	3 (5.6%)	0.661
**NUMBER OF DISEASES** Median (Q1, Q3)	4.00 (3.00, 5.00)	5.00 (4.00, 7.00)	**< 0.001**
**CHARLSON COMORBIDITY INDEX** Median (Q1, Q3)	4.00 (2.00, 6.00)	3.50 (3.00, 6.00)	0.256
**BMI** Median (Q1, Q3)	25.23 (22.04, 28.36)	24.92 (23.03, 30.59)	0.117
**SMOKER**	23 (15.9%)	4 (7.4%)	0.121
N-Miss	1	0	
**MICROBIOTA DYSBIOSIS–RELATED DISEASE**	96 (65.8%)	41 (75.9%)	0.169
DIABETES MELLITUS	45 (46.9%)	20 (48.8%)	0.838
CHOLELITHIASIS	11 (11.5%)	5 (12.2%)	0.902
ISCHAEMIC HEART DISEASE	23 (24.0%)	11 (26.8%)	0.722
AUTOIMMUNE DISEASE	5 (5.2%)	0 (0.0%)	0.137
ASTHMA	10 (10.4%)	5 (12.2%)	0.76
ATOPY	1 (1.0%)	0 (0.0%)	0.512
PSORIASIS	1 (1.0%)	0 (0.0%)	0.512
GASTRIC LYMPHOMA OR CARCINOMA	1 (1.0%)	0 (0.0%)	0.512
COLORECTAL CARCINOMA	6 (6.2%)	3 (7.3%)	0.817
IRRITABLE BOWEL SYNDROME	5 (5.2%)	1 (2.4%)	0.468
INFLAMMATORY BOWEL DISEASE	13 (13.5%)	1 (2.4%)	**0.049**
IDIOPATHIC THROMBOCYTOPENIC PURPURA	1 (1.0%)	0 (0.0%)	0.512
SKIN DISEASE	1 (1.0%)	1 (2.4%)	0.532
PARKINSON DISEASE	6 (6.2%)	4 (9.8%)	0.47
HEPATIC ENCEPHALOPATHY	2 (2.1%)	2 (4.9%)	0.374
BEHAVIOURAL DISORDERS	1 (1.0%)	1 (2.4%)	0.532
COELIAC DISEASE	0 (0.0%)	1 (2.4%)	0.125
ARTHRITIS	3 (3.1%)	5 (12.2%)	**0.038**
IDIOPATHIC CONSTIPATION	1 (1.0%)	0 (0.0%)	0.512
NON-ALCOHOLIC FATTY LIVER	3 (3.1%)	1 (2.4%)	0.827
NUMBER OF MICROBIOTA DYSBIOSIS–RELATED DISEASES Median (Q1, Q3)	1.00 (1.00, 2.00)	1.00 (1.00, 2.00)	0.575
**ALCOHOL INTAKE > 50G/DAY**	8 (5.5%)	1 (1.9%)	0.269
N-Miss	1	0	
**COLECTOMY OR ILEOSTOMY**	15 (10.3%)	5 (9.3%)	0.832
**CHOLECYSTECTOMY**	18 (12.3%)	8 (14.8%)	0.643
**IMMUNOCOMPROMISED**	53 (36.3%)	21 (38.9%)	0.737

Non-Rec, non-recurrent disease; Rec, recurrent disease; N-Miss, number of cases with no information. Q1, Q3, quartile 1, quartile 3. BMI, body mass index. Significant p values (<0.05) are shown in bold.

No significant differences regarding risk factors for CDI were recorded in either of the groups. The main risk factor for developing CDI was antibiotic therapy in the month prior to sampling. In the patients with non-recurrent disease, the most frequent antibiotics were cephalosporins, followed by penicillins, carbapenems and quinolones, while in the patients with recurrent disease the most frequently administered antibiotics were cephalosporins, followed by quinolones, penicillins and carbapenems (**Table 2**). As for other CDI risk factors, treatment with proton pump inhibitors and being hospitalised were widely present (**Table 3**).

**Table 2:** Antibiotic treatment received 1 month prior to the primary *Clostridioides difficile* infection episode.

	Non-Rec (n=131)	Rec (n=51)	p value
**TOTAL DDDs** Median (Q1, Q3)	13.31 (6.00, 22.57)	13.67 (8.00, 22.75)	0.572
**DDDs ANAEROBE** Median (Q1, Q3)	5.71 (2.17, 12.00)	8.79 (2.68, 11.90)	0.44
**SULPHONAMIDE**	18 (13.7%)	4 (7.8%)	0.273
**QUINOLONES**	42 (32.1%)	22 (43.1%)	0.16
**RIFAMYCIN**	11 (8.4%)	2 (3.9%)	0.292
**CARBAPENEMS**	43 (32.8%)	12 (23.5%)	0.22
**GLUCOPEPTIDES**	20 (15.3%)	3 (5.9%)	0.087
**1ST GENERATION CEPHALOSPORINS**	13 (9.9%)	7 (13.7%)	0.461
**2ND GENERATION CEPHALOSPORINS**	6 (4.6%)	3 (5.9%)	0.716
**3RD GENERATION CEPHALOSPORINS**	44 (33.6%)	23 (45.1%)	0.148
**4TH GENERATION CEPHALOSPORINS**	6 (4.6%)	3 (5.9%)	0.716
**5TH GENERATION CEPHALOSPORINS**	3 (2.3%)	0 (0.0%)	0.276
**PENICILLINS**	55 (42.0%)	21 (41.2%)	0.921
**LIPOPEPTIDES**	3 (2.3%)	1 (2.0%)	0.892
**AMINOGLYCOSIDES**	6 (4.6%)	1 (2.0%)	0.409
**PHOSPHONATES**	6 (4.6%)	2 (3.9%)	0.846
**NITROIMIDAZOLE**	16 (12.2%)	8 (15.7%)	0.534
**MACROLIDES**	8 (6.1%)	2 (3.9%)	0.561
**OXAZOLIDINONES**	11 (8.4%)	2 (3.9%)	0.292
**LINCOSAMIDES**	1 (0.8%)	3 (5.9%)	**0.034**
**MONOBACTAMS**	1 (0.8%)	1 (2.0%)	0.486
**TETRACYCLINES**	2 (1.5%)	0 (0.0%)	0.375
**CEPHALOSPORINS**	67 (51.1%)	32 (62.7%)	0.158
**BETALACTAMS**	115 (87.8%)	44 (86.3%)	0.783
**ANAEROBICS**	95 (72.5%)	33 (64.7%)	0.3

Non-Rec, non-recurrent disease; Rec, recurrent disease; N-Miss, number of cases with no information. Q1, Q3, quartile 1, quartile 3. Significant p values (<0.05) are shown in bold.

**Table 3:** Risk factors for *Clostridioides difficile* infection.

	Non-Rec (n=146)	Rec (n=54)	p value
**ANTIFUNGAL TREATMENT**	20 (13.8%)	5 (9.3%)	0.391
N-Miss	1	0	
**ANTIBIOTIC TREATMENT**	131 (89.7%)	51 (94.4%)	0.301
**NUMBER OF ANTIBIOTICS** Median (Q1, Q3)	3.00 (1.00, 4.00)	2.00 (1.00, 4.00)	0.753
**PROTON PUMP INHIBITOR TREATMENT**	121 (83.4%)	45 (83.3%)	0.985
N-Miss	1	0	
**NASOGASTRIC TUBE**	25 (17.2%)	4 (7.4%)	0.08
N-Miss	1	0	
**MECHANICAL VENTILATION**	31 (21.4%)	7 (13.0%)	0.179
N-Miss	1	0	
**SURGERY**	39 (26.9%)	8 (14.8%)	0.074
N-Miss	1	0	
**NEUTROPENIA**	23 (16.4%)	7 (13.2%)	0.581
N-Miss	6	1	
**RISK OF INFECTION NEUTROPENIA**	16 (11.4%)	3 (5.7%)	0.23
N-Miss	6	1	
**CHEMOTHERAPY OR RADIOTHERAPY**	30 (20.7%)	7 (13.0%)	0.213
N-Miss	1	0	
**DIALYSIS**	10 (6.8%)	1 (1.9%)	0.169
**IMMUNOSUPPRESSIVE TREATMENT**	50 (34.5%)	18 (33.3%)	0.879
N-Miss	1	0	

Non-Rec, non-recurrent disease; Rec, recurrent disease; N-Miss, number of cases with no information.Q1, Q3, quartile 1, quartile 3.

Regarding the severity of the CDI episodes, most were mild (non-recurrent 65.8%; recurrent 64.8%; p=0.901). There were 2 cases of toxic megacolon and 2 cases of pseudomembranous colitis, all in the non-recurrent group. Most CDI episodes (non-recurrent 57.5%; recurrent 38.9%; p=0.020) were hospital onset, healthcare facility–associated (**Table 4**). The median toxin B PCR cycle was lower in patients with recurrent disease than in those with non-recurrent disease (23.25 vs 26.10; p=0.003). Those with recurrent disease had higher levels of creatinine and faecal calprotectin than those with non-recurrent disease (all p<0.05) (**Table 4**).

**Table 4:** Characteristics of the primary *Clostridioides difficile* infection episode.

	Non-Rec (n=146)	Rec (n=54)	p value
**EPISODE TYPE**			**0.008**
CA-CDI	32 (21.9%)	10 (18.5%)	0.6
CO-HCFA	26 (17.8%)	17 (31.5%)	**0.037**
HO-HCFA	84 (57.5%)	21 (38.9%)	**0.020**
Undetermined	4 (2.7%)	6 (11.1%)	**0.016**
**TOXIN B PCR CYCLE** Median (Q1, Q3)	26.10 (23.50, 29.90)	23.55 (21.80, 27.12)	**0.003**
N-Miss	5	0	
**TOXIN B PCR CYCLE**			**0.004**
N-Miss	5	0	
<23	29 (20.6%)	22 (40.7%)	
>23	112 (79.4%)	32 (59.3%)	
**BINARY TOXIN**	34 (23.9%)	10 (18.5%)	0.416
N-Miss	4	0	
**HYPERVIRULENT RIBOTYPE**			0.112
No hypervirulent	140 (95.9%)	50 (92.6%)	
181	3 (2.1%)	4 (7.4%)	
027	3 (2.1%)	0 (0.0%)	
**FEVER PREVIOUS 24 H**	42 (28.8%)	13 (24.1%)	0.509
**DAYS OF DIARROHEA** Median (Q1, Q3)	4.00 (2.00, 7.00)	5.00 (4.00, 8.00)	0.018
N-Miss	4	0	
**ABDOMINAL PAIN**	76 (52.1%)	24 (44.4%)	0.339
**ABDOMINAL BLOATING**	28 (19.4%)	7 (13.0%)	0.287
N-Miss	2	0	
**TOXIC MEGACOLON**	2 (1.4%)	0 (0.0%)	0.387
**PSEUDOMEMBRANOUS COLITIS**	2 (1.4%)	0 (0.0%)	0.175
**LEUCOCYTES** Median (Q1, Q3)	8,500.00 (5,970.00, 12,500.00)	10,900.00 (7,110.00, 14,700.00)	0.181
N-Miss	11	3	
**NEUTROPHILS** Median (Q1, Q3)	6,300.00 (3,500.00, 10,050.00)	7,500.00 (4,900.00, 12,600.00)	0.213
N-Miss	11	3	
**ALBUMIN** Median (Q1, Q3)	3.30 (3.00, 3.73)	3.40 (2.90, 4.00)	0.472
N-Miss	74	29	
**Aspartate transaminase (AST)** Median (Q1, Q3)	29.00 (18.75, 78.00)	43.00 (42.00, 50.00)	0.362
N-Miss	114	49	
**Alanine transaminase (ALT)** Median (Q1, Q3)	19.00 (12.00, 33.25)	14.00 (9.50, 22.50)	**0.025**
N-Miss	24	7	
**CREATININE** Median (Q1, Q3)			0.059
N-Miss	10	3	
	0.92 (0.62, 1.48)	1.23 (0.82, 1.67)	
**CREATININE**			**0.003**
N-Miss	10	3	
<1	76 (55.9%)	16 (31.4%)	
>1	60 (44.1%)	35 (68.6%)	
**FAECAL CALPROTECTIN (µg/mg)** Median (Q1, Q3)			**0.01**
N-Miss	18	3	
	92.91 (2.67, 172.25)	150.36 (57.11, 253.75)	
**FAECAL CALPROTECTIN**			**0.001**
N-Miss	18	3	
higher_185	27 (21.1%)	23 (45.1%)	
lower_185	101 (78.9%)	28 (54.9%)	
**EPISODE SEVERITY**			0.156
mild	96 (65.8%)	35 (64.8%)	0.901
severe	37 (25.3%)	18 (33.3%)	0.261
severe-complicated	13 (8.9%)	1 (1.9%)	0.083
**OTHER CAUSE OF DIARRHOEA**	138 (94.5%)	52 (96.3%)	0.609
**CDI TREATMENT RECEIVED**	142 (97.9%)	54 (100.0%)	0.287
N-Miss	1	0	
**METRONIDAZOLE TREATMENT**	50 (34.2%)	14 (25.9%)	0.252
**VANCOMYCIN TREATMENT**	125 (85.6%)	48 (88.8%)	0.617
**FIDAXOMYCIN TREATMENT**	3 (2.1%)	3 (5.6%)	0.201
**FMT**	10 (6.8%)	4 (7.4%)	0.9
**BEZLOTOXUMAB**	9 (6.2%)	1 (1.9%)	0.211

Non-Rec, non-recurrent disease; Rec, recurrent disease; CDI, *Clostridioides difficile* infection; N-Miss, number of cases with no information. Q1, Q3, quartile 1, quartile 3. Significant p values (<0.05) are shown in bold.

In both groups, most patients received treatment for CDI (non-recurrent, 97.9% [142/146]; recurrent, 100% [54/54]), mainly with vancomycin (non-recurrent, 85.6%, recurrent, 88.7%), followed by metronidazole (non-recurrent, 34.2%, recurrent, 26.4%). Fourteen patients included in this study had been enrolled in a clinical trial with faecal microbiota transplantation as their initial treatment. Few patients received fidaxomicin, and few patients received bezlotoxumab. There were no significant differences between the groups with respect to the CDI treatment they received (**Table 4**).

One patient with recurrent disease died (probably CDI-related), and no patients in the non-recurrent group died from a probably CDI-related condition. Both 30-day mortality and 90-day mortality (3.7% and 11.1%, respectively) were higher in the recurrent group than in the non-recurrent group (0% and 1.4% respectively) (all p<0.05) (**Table 5**).

**Table 5:** Outcome of the primary CDI episode.

	Non-Rec (n=146)	Rec (n=54)	p value
**ICU ADMISSION**	6 (4.1%)	1 (1.9%)	0.441
**OUTCOME AT DISCHARGE**			0.113
N-Miss	19	8	
Dead	1 (0.8%)	2 (4.3%)	
Healing	126 (99.2%)	44 (95.7%)	
**CDI-RELATED DEATH**			0.194
Not related	1 (0.7%)	1 (1.9%)	
Probably related	0 (0.0%)	1 (1.9%)	
**TREATMENT FAILURE**	0 (0.0%)	1 (1.9%)	0.103
N-Miss	3	0	
**30-DAY MORTALITY**	0 (0.0%)	2 (3.7%)	**0.019**
**90-DAY MORTALITY**	2 (1.4%)	6 (11.1%)	**0.002**

Non-Rec, non-recurrent disease; Rec, recurrent disease; CDI, *Clostridioides difficile* infection; N-Miss, number of cases with no information.

Probably CDI-related death: Death was considered CDI-related when there were no other attributable causes and/or it occurred within 10 days after the diagnosis of CDI and/or was due to known complications of CDI.

ICU, intensive care unit. Significant p values (<0.05) are shown in bold.

### 3.2 Calprotectin levels and recurrent CDI predictive models

We analyzed 179 samples for calprotectin levels (128 from non-recurrent patients and 51 from recurrent patients). The median values obtained were 98.92µg/mg (IQR 8.33-191.05). We observed significant differences between non-recurrent and recurrent patients (92.91 µg/mg vs 150.36 µg/mg; p=0.01).We obtained a median calprotectin value of 81.32 µg/mg (IQR: 0.00-171.02) for mild CDI episodes and 148.16 µg/mg (IQR: 55.83-230.38) for severe CDI episodes (p=0.002).

We classified faecal calprotectin levels as >185µg/mg and <185µg/mg (45.1% and 21.1% of the recurrent group, respectively; p=0.01) (**Table 6**). Patients with faecal calprotectin >185µg/mg had a lower median toxin B PCR cycle threshold than patients with faecal calprotectin levels <185µg/mg (24.00 vs 25.60; p=0.034). Patients with faecal calprotectin higher than 185µg/mg had higher levels of leucocytes and more days of diarrhoea than patients with lower levels of faecal calprotectin. In addition, more patients had severe and severe-complicated CDI episodes with a calprotectin level >185µg/mg and more mild episodes with calprotectin <185µg/mg (all p<0.05) (**Table 6**).

**Table 6:** Characteristics of patients and *Clostridioides difficile* infection episode according to faecal calprotectin levels.

	Lower than 185 μg/mg (n=129)	Higher than 185 μg/mg (n=50)	p value
**AGE** Median (Q1, Q3)	71.00 (58.00, 80.00)	74.00 (59.25, 84.75)	0.262
**SEX**			0.663
Female	78 (60.5%)	32 (64.0%)	
Male	51 (39.5%)	18 (36.0%)	
**TOXIN A/B POSITIVE (EIA)**	73 (57.5%)	30 (60.0%)	0.76
N-Miss	2	0	
**TOXIN B PCR CYCLE** Median (Q1, Q3)	25.60 (23.05, 29.55)	24.00 (22.20, 27.10)	0.034
N-Miss	2	1	
**BINARY TOXIN POSITIVE (PCR)**	25 (19.5%)	14 (28.6%)	0.194
N-Miss	1	1	
**MICROBIOTA DYSBIOSIS–RELATED DISEASE**	95 (73.6%)	32 (64.0%)	0.202
**IMMUNOCOMPROMISED**	57 (44.2%)	12 (24.0%)	**0.013**
**NEUTROPENIA (< 600/mm^3^)**	16 (12.6%)	0 (0.0%)	**0.012**
N-Miss	2	4	
**DAYS OF DIARROHEA** Median (Q1, Q3)	4.00 (2.00, 7.00)	5.00 (3.00, 9.00)	**0.042**
N-Miss	2	2	
**LEUCOCYTES** Median (Q1, Q3)	8150.00 (5450.00, 11400.00)	11200.00 (7320.00, 16800.00)	**0.006**
N-Miss	9	2	
**EPISODE SEVERITY**			**< 0.001**
Mild	98 (76.0%)	23 (46.0%)	**< 0.001**
Severe	23 (17.8%)	23 (46.0%)	**< 0.001**
Severe-complicated	8 (6.2%)	4 (8.0%)	0.666
**CDI RECURRENCE**	28 (21.7%)	23 (46.0%)	**0.001**
**TOXIN B PCR CYCLE**			0.102
N-Miss	2	1	
<23	31 (24.4%)	18 (36.7%)	0.102
>23	96 (75.6%)	31 (63.3%)	0.102

CDI, *Clostridioides difficile* infection; N-Miss, Number of cases with no information. EIA, enzyme immunoassay. Q1, Q3, quartile 1, quartile 3. Significant p values (<0.05) are shown in bold.

**Figure 1:**
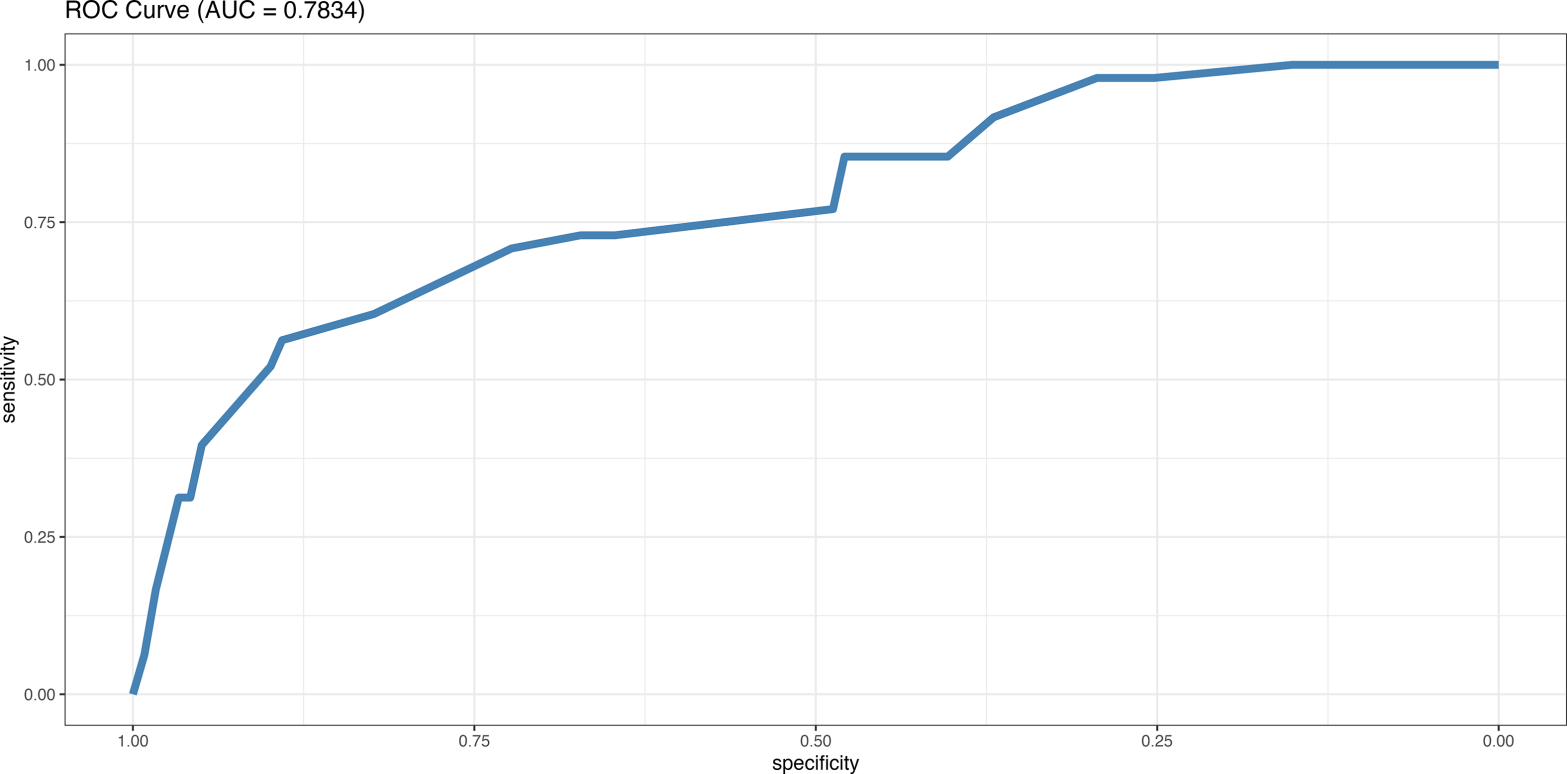
ROC curve (receiver operating characteristic curve) of our model of independent risk factors for developing a recurrent CDI: faecal calprotectin level >185µg/mg, toxin B PCR cycle lower than 23, immunosuppression, female gender, age higher than 65 years, and creatinin levels higher than 1mg/dL (all p<0.05).

We ran a multivariate model and found the following independent risk factors for developing R-CDI: age, faecal calprotectin level >185µg/mg, toxin B PCR cycle threshold <23, immunosuppression, female sex, and creatinine levels higher than 1mg/dL (all p<0.05). We built a risk index for developing R-CDI based on the risk factors we obtained in the multivariable model (**Table 7**). The ROC curve analysis showed that this risk index had an area under the curve (AUC) of 0.783, with a power of 0.999, significance level of 0.05 and an overall accuracy of 79.0% (**Figure 1**).

**Table 7:** Predictive model of recurrent *Clostridioides difficile* infection.

Characteristic	OR*^1^*	95% CI*^1^*	P value	Points
Faecal calprotectin				
>185 µg/mg	4.56	1.91, 11.5	<0.001	4
Toxin B PCR Cycle				
<23	3.33	1.37, 8.42	0.009	3
Immunosuppression				
Yes	5.08	1.96, 14.3	0.001	5
Sex				
Female	4.94	2.01, 13.5	<0.001	5
Creatinine				
>1	3.40	1.45, 8.39	0.006	3
Age				
>70	5.74	2.22, 16.4	<0.001	6

^1^ OR, odds ratio; CI, confidence interval.

### 3.3 Community structure (diversity)

When studying the primary episode microbiome diversity of patients who went on to develop R-CDI and those who did not, we found significant differences in the inverse Simpson index (p=0.021), with lower values in patients with non-recurrent disease.

We found significant differences between those who presented any type of inflammatory bowel disease in the Shannon index, richness and evenness, with lower values than those of patients who did not present this condition (all p<0.05). Patients with a nasogastric tube during the month prior to the episode had lower values in the Shannon index, richness and evenness (all p<0.05).We found lower evenness values for patients who had taken probiotics in the month prior to the episode and those who had undergone colectomy or ileostomy and lower richness values for smokers and patients who had received antifungal treatment in the month prior to the episode.

We also studied the effects of antibiotics on the diversity of the microbiota and found that patients who received more than 3 antibiotics had less alpha diversity, less evenness (all p<0.05) and significant differences in beta diversity with respect to patients who received fewer than 3 antibiotics. The differences in alpha diversity and evenness remained significant in patients with recurrent disease compared with those who had non-recurrent disease.

In terms of beta diversity and of the variance of the intra-group samples, we found no significant differences between the recurrent and the non-recurrent group.

### 3.4 Community composition (relative abundance of taxa)

Regarding the relative abundance of the different taxonomic groups, we found that Bacteroidetes was the major phylum in patients with non-recurrent disease (40.18%), followed by Firmicutes (35.69%), whereas in those with recurrent disease, the major phylum was Firmicutes (38.26%), followed by Bacteroidetes (37.63%). Differences in relative abundance between the groups were not significant.

**Figure 2:**
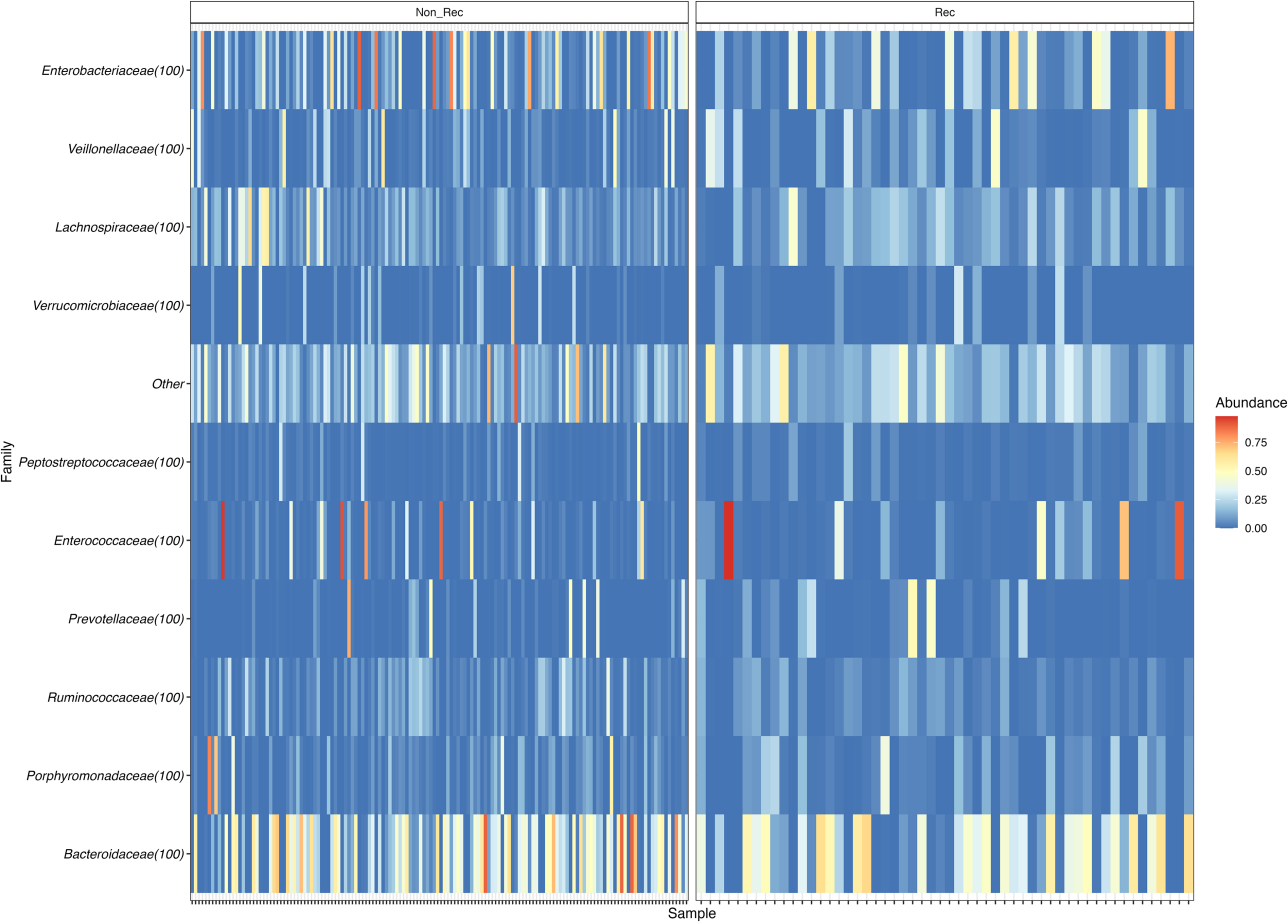
Heatmap of family relative abundance in each sample, divided in Non-recurrent patients (group Non-Rec; left) and recurrent patients (Rec; right).

**Figure 3:**
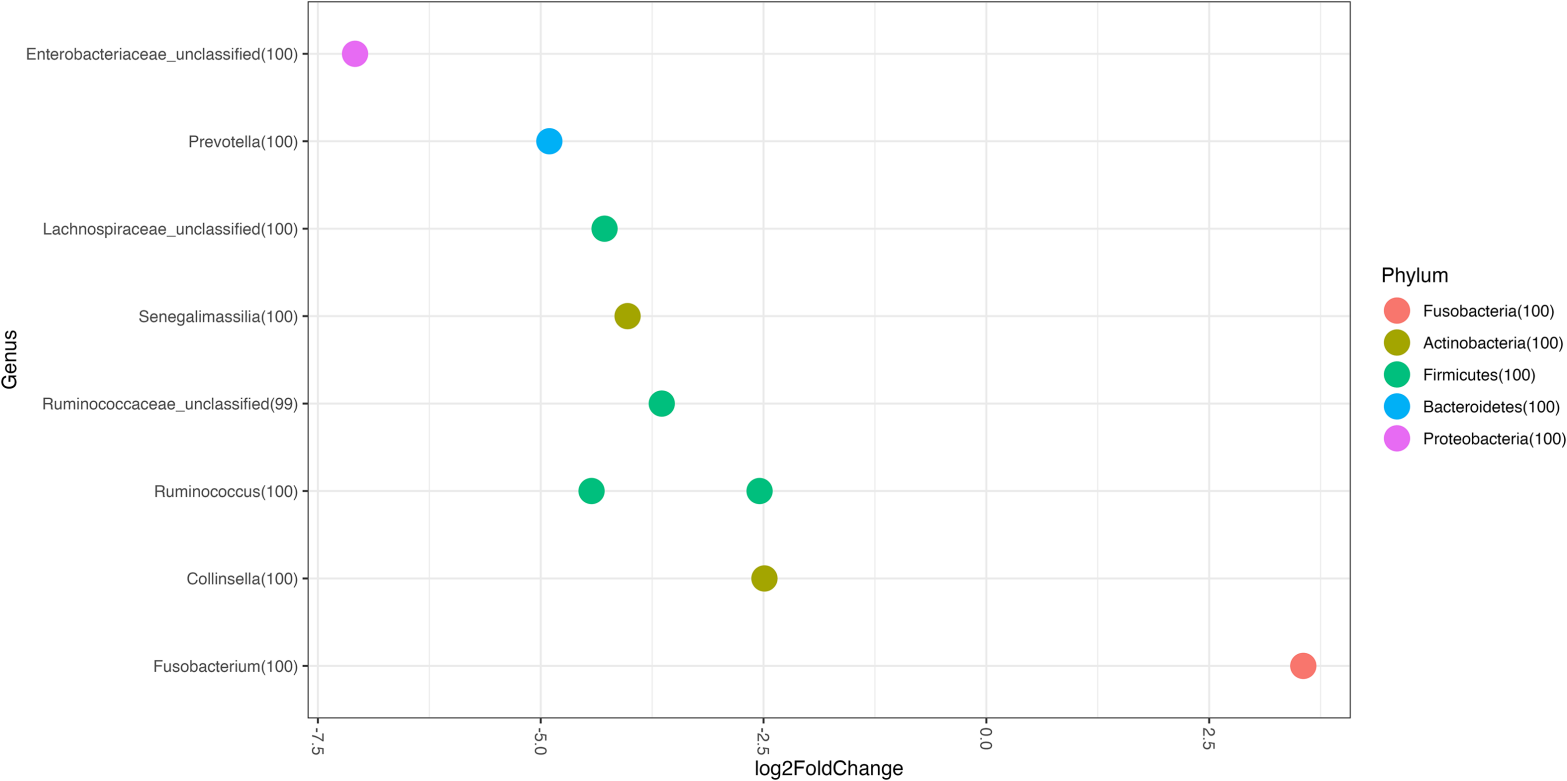
Genus Log2 Fold Change represent differentially abundance genus between groups. Recurrent patients vs Non-recurrent patients.

At the taxonomic family level, we found Bacteroidaceae to be the most abundant taxon in all patients (non-recurrent, 30.57%; recurrent, 28.02%), followed by Enterobacteriaceae (non-recurrent, 16.77%; recurrent, 13.75%) (**Figure 2**). When we analysed the taxonomic level of genus, we found that *Fusobacterium* increased in abundance in recurrent versus non-recurrent disease and that *Collinsella*, *Senegalimassilia*, *Prevotella* and *Ruminococcus* decreased in abundance in recurrent versus non-recurrent disease (all p<0.05) (**Figure 3**).

**Figure 4:**
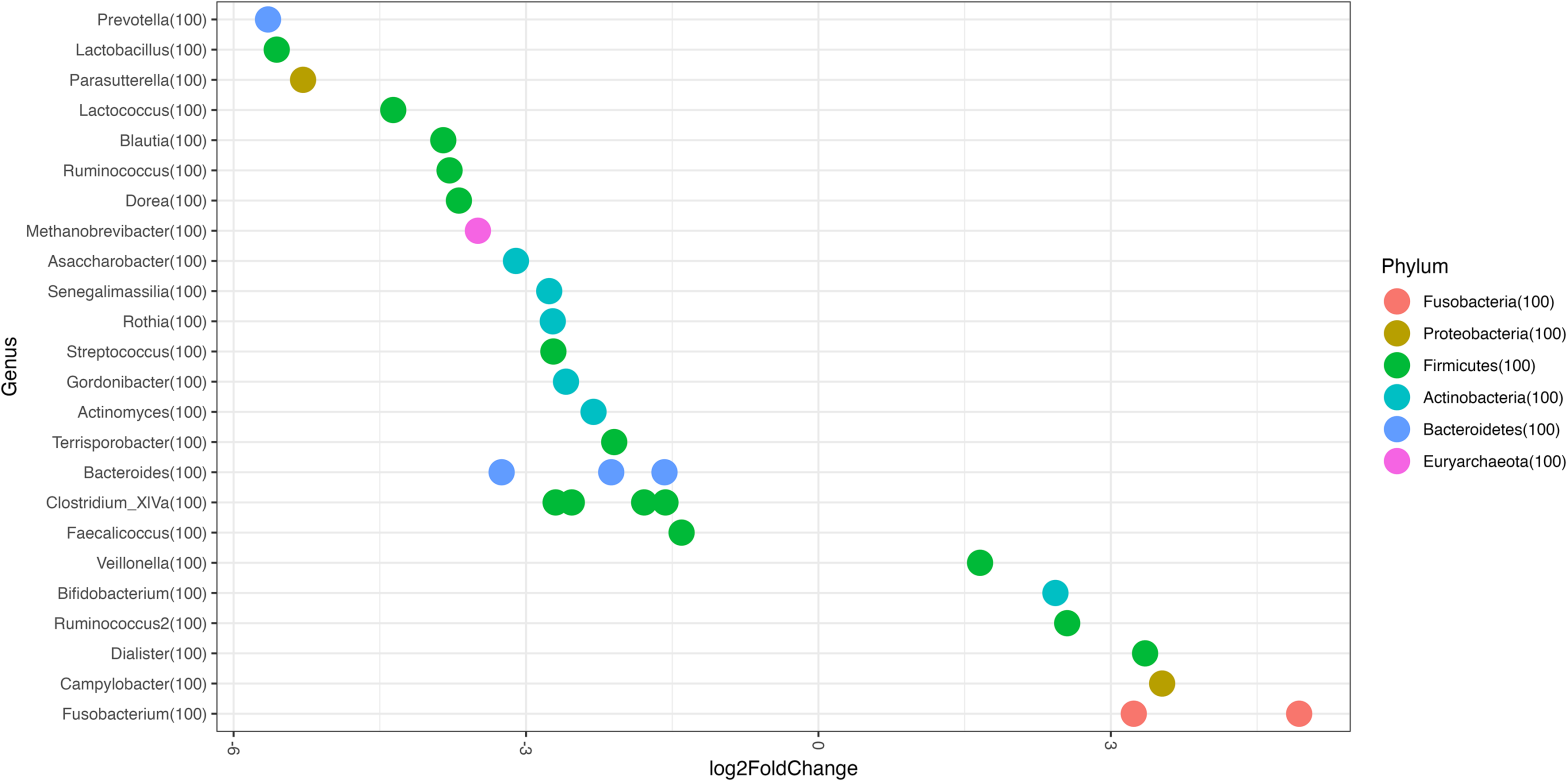
Genus Log2 Fold Change represent differentially abundance genus between the following groups: Fecal calprotectin >185*μ*g/mg vs <185*μ*g/mg.

Our analysis of the differences between patients with calprotectin levels >185µg/mg and those with levels <185µg/mg revealed that, as in the recurrent group, *Fusobacterium* increased and *Senegalimassilia*, *Prevotella* and *Ruminococcus* decreased. We also found other differences such as lower abundance of *Lactobacillus*, *Lactococcus*, *Streptococcus*, *Blautia*, *Dorea, Bacteroides, Faecalicoccus, Clostridium XVIa* and *Parasutterella* in patients with faecal calprotectin >185µg/mg than in those with lower values (all p<0.05) (**Figure 4**).

We performed a multivariable association study between microbial communities and clinical metadata and found that the genera *Senegalimassilia, Alistipes, Blautia* and *Lactococcus* had a negative association with faecal calprotectin concentration and that the genera *Bacteroides, Fusobacterium* and *Dialister* had a positive association with faecal calprotectin concentration (all p<0.05) (**Table 8**).

**Table 8:** Association between microbial communities and calprotectin concentrations.

Genus	Coefficient	Standard deviation	p value	q value
*Senegalimassilia*	-2.451	0.7983	0.0025	0.043
*Bacteroides*	3.136	0.6656	<0.001	<0.001
*Coprobacter*	1.756	0.4819	<0.001	0.013
*Alistipes*	-1.723	0.5023	<0.001	0.020
*Lactococcus*	-4.241	0.9881	<0.001	0.002
*Finegoldia*	2.760	0.5526	<0.001	<0.001
*Blautia*	-2.854	0.7209	<0.001	0.005
*Coprococcus*	-2.219	0.6547	<0.001	0.022
*Faecalibacterium*	-4.352	0.8891	<0.001	<0.001
*Dialister*	2.839	0.4885	<0.001	<0.001
*Megasphaera*	6.351	0.8040	<0.001	<0.001
*Fusobacterium*	2.141	0.5655	<0.001	0.009
*Akkermansia*	-2.184	0.5800	<0.001	0.009

Bacterial genera positively or negatively associated with faecal calprotectin concentrations.

In terms of similarities between the OTUs of both groups, we found that they shared 1921 OTUs: 1119 unique OTUs in the recurrent group and 2241 unique OTUs in the non-recurrent group.

## 4 Discussion

In the present study, we profiled the microbiota of a large cohort of patients with CDI and found marked differences that could be useful in predicting R-CDI. We also established a significant association between elevated calprotectin values in patients who will subsequently develop R-CDI and built a risk index for R-CDI.

Several studies have characterised the microbiota in patients with CDI and R-CDI, although very few have attempted to establish relationships between gut microbiota and the likelihood of developing R-CDI (Khanna et al., 2016; Seekatz et al., 2016; Pakpour et al., 2017; Dawkins et al., 2022). These studies include a limited number of patients, and their results are conflicting.

With respect to diversity, we found significant differences in alpha diversity (inverse Simpson index) between patients who later developed R-CDI and those who did not. This was also observed in a smaller study by Pakpour et al. (Pakpour et al., 2017). When studying beta diversity, we did not observe any significant differences between the recurrent and non-recurrent groups, consistent with findings published elsewhere, except for those of Pakpour et al. who found significant differences in beta diversity between these groups (Khanna et al., 2016; Seekatz et al., 2016; Pakpour et al., 2017; Dawkins et al., 2022).

With respect to changes in genus abundance, we observed significant differences in genera such as *Fusobacterium*, which is more abundant in recurrent than non-recurrent disease, and in *Collinsella*, *Senegalimassilia*, *Prevotella* and *Ruminococcus*, with lower abundance in recurrent than in non-recurrent disease. Very few data have been reported on taxa abundance in patients with primary CDI who later develop R-CDI, and the available data are conflicting (Khanna et al., 2016; Seekatz et al., 2016; Pakpour et al., 2017; Dawkins et al., 2022).

The genus *Fusobacterium*, which we observed to have higher abundance in patients who will further develop R-CDI, is considered an opportunistic pathogen associated with a wide range of human conditions, including inflammatory bowel disease, colorectal cancer, preterm birth and intestinal inflammation (Strauss et al., 2011; Amitay et al., 2017; Engevik et al., 2021). The genera that we found in lower abundance in patients with recurrent disease, such as *Collinsella*, *Senegalimassilia*, *Prevotella* and *Ruminococcus*, were previously associated with healthy individuals, had anti-inflammatory properties and produced metabolites that are very important for the preservation of the intestinal barrier (Ze et al., 2012; Ko et al., 2017; Adamberg et al., 2018; Yeoh et al., 2022).

The devalences of these genera could play a role in maintaining intestinal inflammation and thus providing a favourable environment for new R-CDI, although this has not been demonstrated.

These microbiota findings may relate to the results we had obtained for calprotectin, with higher levels in recurrent disease than in non-recurrent disease, as calprotectin is a good biomarker of intestinal inflammation. We also found the same response for some of the above-mentioned genera in patients with high calprotectin levels than in those with low calprotectin levels. In this sense, at diagnosis, patients who are likely to develop R-CDI have higher levels of calprotectin, together with a higher abundance of *Fusobacterium* and lower abundance of *Senegalimassilia*, *Prevotella* and *Ruminococcus*. To the best of our knowledge, this is the first study to report an association between calprotectin levels and microbiota abundance alterations (in CDI).

However, although it is known that faecal calprotectin reaches the gut via migration of neutrophils into gastrointestinal tissues due to inflammatory processes, the exact mechanism by which this happens, and the genera or species of microbes involved in this process, are not known (Pathirana et al., 2018).

In the literature, some studies use calprotectin levels to distinguish true episodes of CDI from colonisation by *C. difficile* (Popiel et al., 2015) or to classify the severity of the CDI episode. We obtained statistically significantly higher levels of faecal calprotectin in severe episodes than in mild episodes, consistent with previous studies, which reported significant results between calprotectin levels and the severity of the CDI episode. (Barbut et al., 2017; Kim et al., 2017; Han et al., 2020; Suarez-Carantoña et al., 2021). However, few studies have investigated the relationship between calprotectin levels and risk of developing R-CDI: those that did showed no significant association between R-CDI and calprotectin levels, and most of them included a low number of patients studied or only included a specific subpopulation such as children or cancer patients (Swale et al., 2014; Peretz et al., 2016; Nicholson et al., 2017; He et al., 2018). Our results for calprotectin reveal a significant association between elevated calprotectin values in patients who will subsequently develop R-CDI, possibly because we analysed a large cohort of patients.

We propose an early prediction model for R-CDI based on clinical and analytical data such as age, sex, immunosuppression, creatinine level, calprotectin level and *C. difficile* PCR toxin B gene cycle, which can be assessed using commercially available PCR methods found in many microbiology laboratories. Our R-CDI prediction model included variables already known to be risk factors for R-CDI or poor outcome of CDI, such as advanced age, female sex, creatinine levels >1mg/dL, *C. difficile* PCR toxin B gene cycle threshold <23, immunosuppression and faecal calprotectin levels (Hu et al., 2009; Eyre et al., 2012; Lupse et al., 2013; Miller et al., 2013; Reigadas et al., 2016; Origüen et al., 2019; Alrahmany et al., 2021).

Hence, the data on which the model is based are easy to obtain at the time of diagnosis of the primary CDI episode, with a reasonable AUC (0.783). Although several prediction models have been developed for R-CDI (Eyre et al., 2012; D’Agostino et al., 2014; LaBarbera et al., 2015; Escobar et al., 2017; Cobo et al., 2018; Ruzicka et al., 2022), none have been widely implemented in clinical practice. External validations of prediction tools have revealed disappointing results, probably owing to the low quality of most studies and the small effects of the proposed prognostic factors (van Rossen et al., 2022).

Most of these predictive R-CDI models yielded much lower AUC values than ours, including those of Ruzicka et al. (AUC 0.65-0.63), Cobo et al. (AUC 0.72), Zilberberg et al. (AUC 0.643), Escobar et al. (AUC 0.591-0.605), D’Agostino et al. (AUC 0.64) and Larrainzar-Coghen et al. (AUC 0.66). Of these models, only that of Cobo et al. incorporated laboratory parameters related to *C. difficile* (D’Agostino et al., 2014; Larrainzar-Coghen et al., 2016; Zilberberg et al., 2016; Escobar et al., 2017; Cobo et al., 2018; Ruzicka et al., 2022).Origüen et al. obtained a predictive model for R-CDI with an AUC very similar to ours, ie, 0.785. Their model also included the PCR cycles of the *C. difficile* toxin (as did ours), although when they included the result of toxin detection by EIA, their AUC dropped to 0.775 (Origüen et al., 2019). We found only 2 models with higher AUC values than ours, namely, that of Hu et al. (AUC 0.80 in the validation cohort, 0.62 in the derivation cohort), which includes the detection of *C. difficile* antitoxin A IgG, and that of LaBarbera et al. (AUC 0.83), which does not include any laboratory parameters (Hu et al., 2009; LaBarbera et al., 2015). However, given that the model of Hu et al. is based on obtaining antitoxin IgA levels at day 12 of the CDI diagnosis, it cannot be used to guide treatment at the beginning of the episode. The model of LaBarbera et al. was complex, including 25 different variables with an overall accuracy of 66.1%, whereas ours was 79%. Having a simple and good R-CDI prediction model could help to guide early treatment of R-CDI.

Our study and our prediction model are limited by their single-centre design; therefore, our results should be validated in multicentre studies. On the other hand, we recruited a large number of patients, making ours the largest cohort to date in which microbiota in CDI and risk of recurrence are analysed. Our results can therefore be considered robust.

In conclusion, abundance of various bacterial genera and faecal calprotectin levels could be used as biomarkers to help identify patients who will develop R-CDI from those who will not. We also propose the use of our simple R-CDI risk index, which includes readily available data and rapid laboratory tests. The use of these early markers of R-CDI can guide management and treatment of the initial CDI episode, thus optimizing the use of resources, especially for patients who are at increased risk of R-CDI. Further research is warranted.

## HGUGM Microbiome Group

Carmen Moreno, Mara Perellada, Celso Arando, Lorena Cussó, Manuel Desco, Javier Vaquero, Marta Fernández, Rafael Correa, Darío García de Viedma, Ana I. Fernández, Nuria López, Silvia Vázquez-Cuesta, Elena Reigadas, Javier Bermejo, Francisco Fernández-Avilés.

## Data availability statement

The datasets presented in this study can be found in online repositories. The names of the repository/repositories and accession number(s) can be found below: https://www.ebi.ac.uk/ena, PRJEB57947.

## Ethics statement

The studies involving human participants were reviewed and approved by Ethics Committee of Hospital General Universitario Gregorio Marañón in Madrid (number MICRO.HGUGM.2016-029). The patients/participants provided their written informed consent to participate in this study.

## Author contributions

ER and EB designed the study. SV-C collected data and samples. SV-C and NG performed the data analysis. SV-C, ER, EB, MO, MK, LA, MM and PM contributed to data interpretation. SV-C wrote the first draft of the manuscript. SV-C, ER, EB, MO, MK, LA, MM, JB, FD, PM and AF contributed to the intellectual content and approved the final draft. SV-C, ER and EB had access to and verified the underlying study data. All authors had full access to all the data in the study and had final responsibility for the decision to submit for publication. All authors contributed to the article and approved the submitted version.
