# Supplementary material for: Microbiome profile and calprotectin levels as markers of risk of recurrent Clostridioides difficile infection
Source: Front Cell Infect Microbiol. 2023 Sep 13;13:1237500. doi: 10.3389/fcimb.2023.1237500 (PMC10534046; doi:10.3389/fcimb.2023.1237500)
Supplement: Supplementary file 1 [file Presentation_1.pptx]

## Slide 1
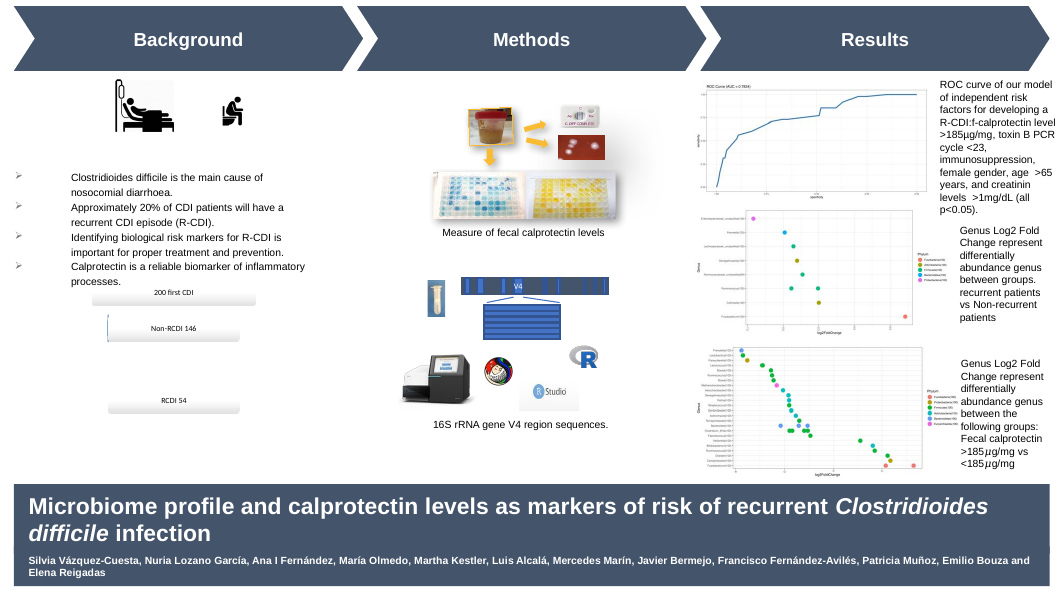

Background
Methods
Results
ROC curve of our model of independent risk factors for developing a R-CDI:f-calprotectin level >185µg/mg, toxin B PCR cycle <23, immunosuppression, female gender, age >65 years, and creatinin levels >1mg/dL (all p<0.05).
Clostridioides difficile is the main cause of nosocomial diarrhoea.
Approximately 20% of CDI patients will have a recurrent CDI episode (R-CDI).
Identifying biological risk markers for R-CDI is important for proper treatment and prevention.
Calprotectin is a reliable biomarker of inflammatory processes.
Genus Log2 Fold Change represent differentially abundance genus between groups. recurrent patients vs Non-recurrent patients
Measure of fecal calprotectin levels
V4
Genus Log2 Fold Change represent differentially abundance genus between the following groups: Fecal calprotectin >185𝜇g/mg vs <185𝜇g/mg
16S rRNA gene V4 region sequences.
Microbiome profile and calprotectin levels as markers of risk of recurrent Clostridioides difficile infection
Silvia Vázquez-Cuesta, Nuria Lozano García, Ana I Fernández, María Olmedo, Martha Kestler, Luis Alcalá, Mercedes Marín, Javier Bermejo, Francisco Fernández-Avilés, Patricia Muñoz, Emilio Bouza and Elena Reigadas
