# Supplementary material for: Microbiome profile and calprotectin levels as markers of risk of recurrent Clostridioides difficile infection
Source: Front Cell Infect Microbiol. 2023 Sep 13;13:1237500. doi: 10.3389/fcimb.2023.1237500 (PMC10534046; doi:10.3389/fcimb.2023.1237500)
Supplement: Supplementary file 2 [file Table_1.docx]

Supplementary Material

Microbiome profile and calprotectin levels as markers of risk of recurrent Clostridioides difficile infection.

Silvia Vázquez-Cuesta^1,2,3*^, Nuria Lozano García^1,2^, Ana I Fernández^2,4^, María Olmedo ^1,2^, Martha Kestler^1,2,5,^,LuisAlcalá, ^1,2,6^, Mercedes Marín^1,2,5,6^, Javier Bermejo^2,4,5,7^, Francisco Fernández-Avilés Díaz^2,4,5,7^, Patricia Muñoz^1,2,5,6^, Emilio Bouza ^1,2,5,6^ and Elena Reigadas^1,2,5^,on behalf of HGUGM Microbiome Group

*** Correspondence:** Silvia Vázquez-Cuesta: silviavazquez.lab@gmail.com

# Supplementary Figures and Tables

Supplementary table 1. Genus Log2 Fold between fecal calprotectin >185𝜇g/mg vs <185𝜇g/mg

| Genus | log2 Fold Change | P adj |
| --- | --- | --- |
| Prevotella(100) | -5.6468 | 1.51E-13 |
| Lactobacillus(100) | -5.5577 | 8.76E-06 |
| Parasutterella(100) | -5.2874 | 7.09E-05 |
| Lactococcus(100) | -4.3621 | 1.11E-03 |
| Blautia(100) | -3.8489 | 7.23E-05 |
| Ruminococcus(100) | -3.7845 | 3.00E-03 |
| Dorea(100) | -3.6885 | 1.28E-02 |
| Methanobrevibacter(100) | -3.4935 | 3.00E-03 |
| Asaccharobacter(100) | -3.1032 | 1.28E-02 |
| Senegalimassilia(100) | -2.7622 | 3.14E-02 |
| Rothia(100) | -2.7255 | 3.43E-03 |
| Streptococcus(100) | -2.7193 | 1.02E-06 |
| Gordonibacter(100) | -2.5900 | 1.28E-02 |
| Actinomyces(100) | -2.3075 | 2.97E-02 |
| Terrisporobacter(100) | -2.0952 | 4.48E-02 |
| Bacteroides(100) | -3.2503 | 1.26E-02 |
| Bacteroides(100) | -2.1260 | 4.02E-02 |
| Bacteroides(100) | -1.5796 | 2.18E-02 |
| Clostridium_XlVa(100) | -2.6966 | 3.44E-02 |
| Clostridium_XlVa(100) | -2.5306 | 2.10E-03 |
| Clostridium_XlVa(100) | -1.7883 | 3.09E-03 |
| Clostridium_XlVa(100) | -1.5696 | 3.48E-02 |
| Faecalicoccus(100) | -1.4041 | 1.29E-02 |
| Veillonella(100) | 1.6578 | 2.18E-02 |
| Bifidobacterium(100) | 2.4310 | 5.86E-03 |
| Ruminococcus2(100) | 2.5517 | 2.63E-02 |
| Fusobacterium(100) | 3.2349 | 4.30E-03 |
| Dialister(100) | 3.3509 | 1.64E-03 |
| Campylobacter(100) | 3.5258 | 1.28E-02 |
| Fusobacterium(100) | 4.9314 | 1.93E-05 |

Genus Log2 Fold Change represent differentially abundance genus between the following groups: Fecal calprotectin >185𝜇g/mg vs <185𝜇g/mg

Supplementary graphical abstract is in another file (pptx format)
